# Individualized Prediction of Postoperative Survival in Gallbladder Cancer: A Nomogram Based on SEER Data and External Validation

**DOI:** 10.3390/cancers17121919

**Published:** 2025-06-09

**Authors:** Yayue Liu, Kangwei Zhu, Xindi Tian, Ping Chen, Qingqing Xiong, Guangtao Li, Xiaochen Ma, Ruyu Han, Liyu Sun, Yijian Shen, Fengyi Zhu, Yimeng Wang, Lu Chen, Tianqiang Song

**Affiliations:** 1 Tianjin Medical University Cancer Institute and Hospital, Tianjin 300060, China; dr_lyy@163.com (Y.L.); jayzhutmu@163.com (K.Z.); 18341672441@163.com (X.T.); chenping@tjmuch.com (P.C.); xiongqingqing@tmu.edu.cn (Q.X.); liguangtaohj@126.com (G.L.); maxiaochen1993@foxmail.com (X.M.); h20216021057@163.com (R.H.); sly178604@126.com (L.S.); shenyijian809@163.com (Y.S.); 15522107871@163.com (F.Z.); wangyimeng2674@163.com (Y.W.); 2 National Clinical Research Center for Cancer, Tianjin 300060, China; 3 Liver Cancer Center, Tianjin 300060, China; 4 Tianjin’s Clinical Research Center for Cancer, Tianjin 300060, China; 5 Tianjin Key Laboratory of Digestive Cancer, Tianjin 300060, China

**Keywords:** gallbladder cancer, nomogram, adjuvant chemotherapy, biliary carcinoma Lymphadenectomy

## Abstract

Background: Gallbladder cancer (GBC) is a rare but aggressive malignancy. Prognostic tools are essential for optimizing postoperative treatment strategies. We aim to develop and validate a prognostic nomogram to estimate 1-, 3-, and 5-year overall survival (OS) in GBC patients and explore the role of adjuvant chemotherapy across different subgroups. Methods: A total of 1848 postoperative GBC patients from the SEER database (2000–2020 17 regions) were analyzed, with an additional external validation cohort of 108 patients from China (2010–2020). Prognostic factors were identified using LASSO regression and multivariable Cox analysis. A nomogram was constructed and validated using the concordance index (C-index), time-dependent ROC curves, calibration curves, and decision curve analysis (DCA). Subgroup analyses were performed to evaluate the impact of adjuvant chemotherapy. Results: The nomogram demonstrated strong predictive performance, with C-indices of 0.767 (training), 0.798 (internal validation), and 0.750 (external validation). Time-dependent ROC curves in the training cohort showed AUCs of 0.777, 0.769, and 0.800 for 1-, 3-, and 5-year OS, respectively. In the internal validation cohort, the corresponding AUCs were 0.763, 0.743, and 0.803. External validation using the independent Chinese cohort of 108 patients showed consistent results, with AUCs of 0.771, 0.835, and 0.810 for 1-, 3-, and 5-year OS. Subgroup analysis revealed that adjuvant chemotherapy significantly improved survival in patients with TNM stage >IIB. In contrast, patients with early-stage disease (TNM ≤ IIB) showed no significant survival benefit from chemotherapy. Conclusions: This study developed a validated prognostic nomogram for postoperative GBC patients, demonstrating strong discrimination and calibration. Subgroup analysis suggests that adjuvant chemotherapy benefits select high-risk patients, aiding personalized decision-making in clinical practice.

## Simple Summary

Gallbladder cancer (GBC) is a rare but aggressive malignancy with poor prognosis. Accurate prognostic tools are essential for guiding postoperative treatment strategies. In this study, we developed and externally validated a prognostic nomogram, demonstrating strong generalizability across both population-based and independent real-world cohorts, aiming to estimate the 1-, 3-, and 5-year overall survival of patients after gallbladder cancer surgery. The model demonstrated high accuracy and clinical utility. Furthermore, subgroup analyses indicated that adjuvant chemotherapy may improve survival in patients with advanced-stage disease. This tool can assist clinicians in making individualized treatment decisions and follow-up plans.

## 1. Introduction

Gallbladder cancer (GBC) is a rare but highly aggressive malignancy and constitutes a significant portion of biliary tract cancers [1,2,3]. Due to its insidious onset and lack of early symptoms, most patients are diagnosed at an advanced stage, often with poor prognosis. A proportion of cases are identified incidentally during or after surgery, further complicating diagnosis and management [4,5]. In addition, the incidence and mortality rates of GBC show notable geographic and ethnic variability, with particularly high burdens reported in, for example, Mapuche Indians, Alaska Natives, and Hispanics, which have been reported to exhibit some of the highest incidence rates of gallbladder cancer worldwide [6]. This variation reflects the influence of regional risk factors such as gallstone prevalence, chronic inflammation, and genetic predispositions [7]. Radical surgical resection is considered the only potentially curative approach for early-stage disease, but the mortality rate remains high even after complete resection [8,9].

The current American Joint Committee on Cancer (AJCC) TNM staging system is widely used to assess prognosis and guide treatment decisions [9,10]. However, it fails to capture many individual-level factors that can influence survival, such as tumor grade, lymph node dissection, and receipt of adjuvant chemotherapy [2]. These limitations pose a challenge for clinicians seeking to make evidence-based decisions tailored to individual patients.

Recent studies, including the BILCAP trial, have highlighted the potential benefit of adjuvant chemotherapy [11]. Meanwhile, nomograms have emerged as practical tools to integrate multiple prognostic variables into individualized survival estimates, offering superior predictive accuracy compared to TNM staging alone. Despite their growing adoption in other cancers [12,13], and although several nomogram-based models for GBC have been developed in recent years, they remain limited in number [3,14,15]. Furthermore, few studies have investigated whether adjuvant chemotherapy provides uniform benefit across TNM-defined subgroups in a real-world population [11,16].

Given the limitations of existing staging systems and the increasing need for tools that support individualized postoperative treatment planning, we designed a study to develop and validate a prognostic nomogram based on a large, population-based cohort of postoperative GBC patients. An independent external dataset was used to confirm the model’s generalizability. In addition, we conducted subgroup analyses to explore whether the potential benefit of adjuvant chemotherapy varies across different TNM stages. Our goal was to provide a clinically useful tool for risk stratification and personalized decision-making in the management of GBC.

## 2. Methods

### 2.1. Study Design

This was a retrospective cohort study based on population-level data from the Surveillance, Epidemiology, and End Results (SEER) database (2000–2020 17 registries) and a single-center cohort from China used for external validation. This study aimed to construct and validate a prognostic nomogram predicting 1-, 3-, and 5-year overall survival (OS) for postoperative GBC patients.

### 2.2. Participants

SEER Cohort: Patients diagnosed with GBC who underwent surgical resection were identified from 17 SEER registries. Inclusion criteria were the following: (1) pathologically confirmed GBC; (2) age ≥ 20 years; (3) receipt of surgery with available survival data; (4) staging available under AJCC 8th edition or mappable from 7th edition. Exclusion criteria included the following: metastatic disease (M1), death due to non-cancer causes, missing staging or outcome data, and loss to follow-up. IVB patients at the TNM stage were excluded from modeling due to singular matrix issues. After exclusions, 1848 patients were included.

External Cohort: An independent cohort of 108 postoperative GBC patients from a single Chinese cancer hospital was used for external validation, using criteria consistent with the SEER population.

### 2.3. Materials

Key variables extracted included demographic data (age, sex, race, marital status, income, region), tumor characteristics (histological grade, AJCC 8th TNM stage), and treatment factors (lymphadenectomy, chemotherapy, radiotherapy). Age was reclassified as follows: <60, 60–70, 70–80, >80 years. TNM stages were consolidated into clinical stages I–IVB per AJCC 8th edition.

### 2.4. Procedure

Missing values for variables such as grade, income, marital status, and residence were imputed using random forest (missForest package in R version 4.4.0, R Foundation for Statistical Computing, Vienna, Austria) [17]. Income was later excluded due to excessive missingness. Patients from the SEER dataset were randomly split 1:1 into training (n = 924) and internal validation cohorts (n = 924). The Chinese cohort (n = 108) served as the external validation set.

**Figure 1:**
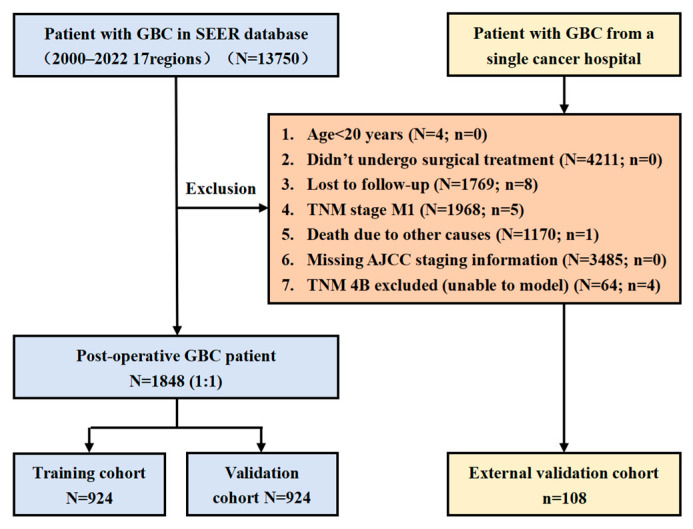
Study flowchart.

The detailed patient selection process is illustrated in Figure 1.

### 2.5. Data Analysis

Descriptive statistics were used to compare baseline characteristics between cohorts. As all variables were categorical, comparisons were conducted using the chi-square test.

Feature selection was performed using least absolute shrinkage and selection operator (LASSO) regression with 10-fold cross-validation to reduce model overfitting [18,19]. The optimal value of the regularization parameter λ was chosen by identifying the minimum mean cross-validated error. Variables with non-zero coefficients in the LASSO model were retained for multivariate modeling.

A multivariate Cox proportional hazards model was then fitted using the selected variables. The hazard ratios (HRs), 95% confidence intervals (CIs), and *p*-values were reported to quantify the strength of association between covariates and overall survival (OS). The proportional hazards assumption was tested using Schoenfeld residuals.

Model performance was assessed using the concordance index (C-index), time-dependent receiver operating characteristic (ROC) curves, calibration curves, and decision curve analysis (DCA).

Based on the final Cox model, a nomogram was constructed to estimate the 1-, 3-, and 5-year OS probabilities. Each predictor was assigned a point value in the nomogram, and total point scores were translated into individualized survival probabilities.

### 2.6. Subgroup Analysis

To further evaluate the heterogeneity of treatment effects, subgroup analyses were performed to explore the potential survival benefit of adjuvant chemotherapy across predefined patient subsets, including demographic factors (age groups, sex, marital status, income level, and geographic region), tumor characteristics (TNM stage, tumor grade), and treatment variables (lymphadenectomy status). Within each subgroup, a separate multivariate Cox proportional hazards model was applied to estimate hazard ratios (HRs) and 95% confidence intervals (CIs) for the effect of chemotherapy, with results visually presented using forest plots. HRs less than 1 were interpreted as indicative of survival benefit, while HRs greater than 1 suggested limited benefit or potential harm. These analyses aimed to help identify patient populations most likely to benefit from chemotherapy and inform personalized treatment strategies.

## 3. Results

### 3.1. Patient Characteristics

A total of 1848 gallbladder cancer patients from the SEER database (2000–2020) and 108 patients from an independent external Chinese cohort were included. The SEER cohort was randomly split into a training set (n = 924) and an internal validation set (n = 924), while the external validation cohort (n = 108) was analyzed separately. Baseline characteristics, including age, sex, TNM stage, lymphadenectomy status, histological grade, and chemotherapy status, were comparable across the training, internal validation, and external validation cohorts. The results show no significant differences between the two groups for most variables; however, marital status exhibited a statistically significant difference (*p* < 0.05), indicating that the randomization successfully balanced key clinical variables (Table 1). Missing values were handled using random forest imputation, except for income, which had excessive missing data and was excluded from further analysis, variable selection, and nomogram construction.

### 3.2. LASSO Regression for Variable Selection

**Figure 2:**
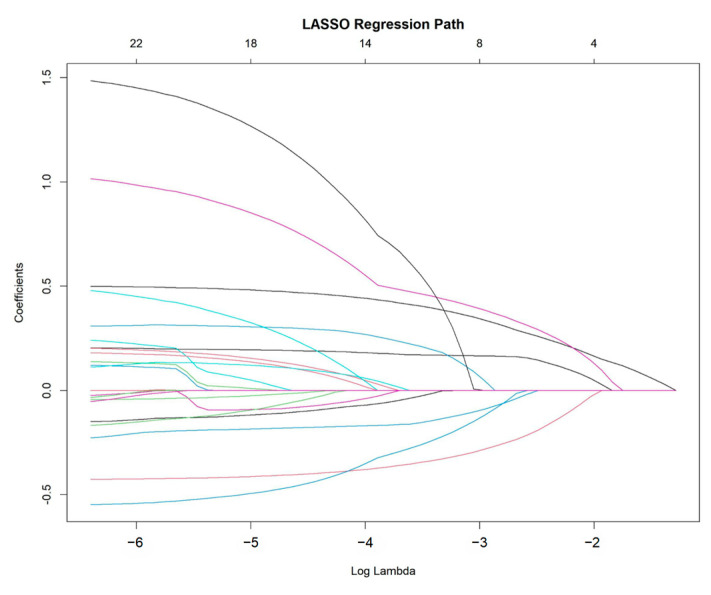
LASSO regression path for variable selection.

**Figure 3:**
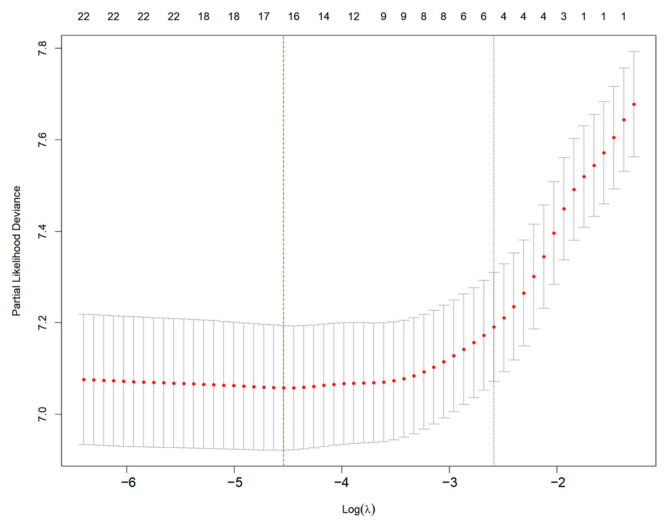
Selection of optimal λ using cross-validation in LASSO regression. Red dots represent partial likelihood deviance from 10-fold cross-validation. The left vertical red dashed line indicates the λ with minimum deviance; the right vertical dotted line indicates the 1-SE criterion.

To identify the most critical prognostic factors, least absolute shrinkage and selection operator (LASSO) regression was applied to the dataset (Figure 2 and Figure 3). This method, known for its ability to handle high-dimensional data and prevent overfitting, was used to refine the selection of variables by shrinking the coefficients of less relevant predictors toward zero. By applying cross-validation, the optimal lambda value was determined, ensuring that only the most significant variables with predictive value were retained in the final model.

### 3.3. Multivariable Cox Regression Modeling

Following LASSO regression, a multivariable Cox proportional hazards model was constructed to further evaluate the independent prognostic impact of the selected variables. The final model incorporated key factors, including age, TNM stage, lymphadenectomy status, histological grade, and chemotherapy status, as they demonstrated significant associations with overall survival in the univariate and LASSO analyses. The Cox model was used to estimate hazard ratios (HRs) and 95% confidence intervals (CIs) for each variable, providing a comprehensive understanding of their relative contributions to patient prognosis. The detailed results of the multivariable Cox regression analysis are presented in Table 2.

### 3.4. Development of a Prognostic Nomogram

**Figure 4:**
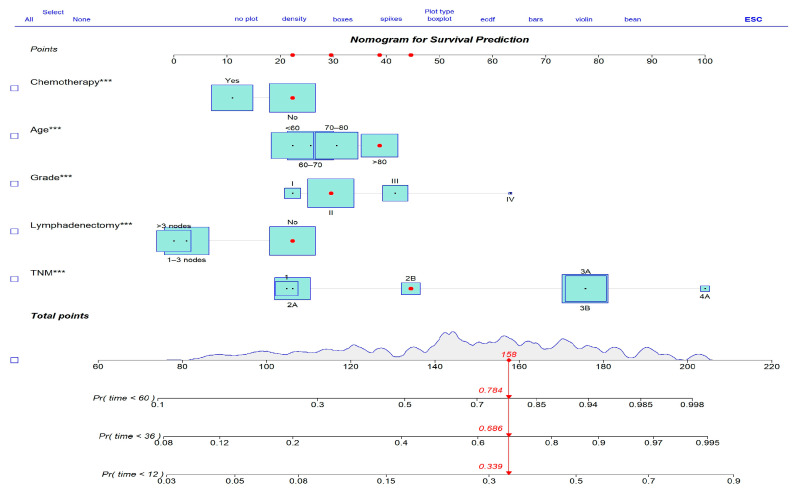
Prognostic nomogram for overall survival prediction. Variables with *** indicate statistical significance at *p* < 0.001.

Based on the final Cox model, a prognostic nomogram was developed to predict 1-, 3-, and 5-year overall survival (OS) for gallbladder cancer patients (Figure 4). The nomogram assigns a weighted score to each independent prognostic factor, allowing clinicians to easily estimate individualized survival probabilities. This tool enhances personalized risk assessment and supports decision-making regarding treatment strategies.

### 3.5. Receiver Operating Characteristic (ROC) Analysis

**Figure 5:**
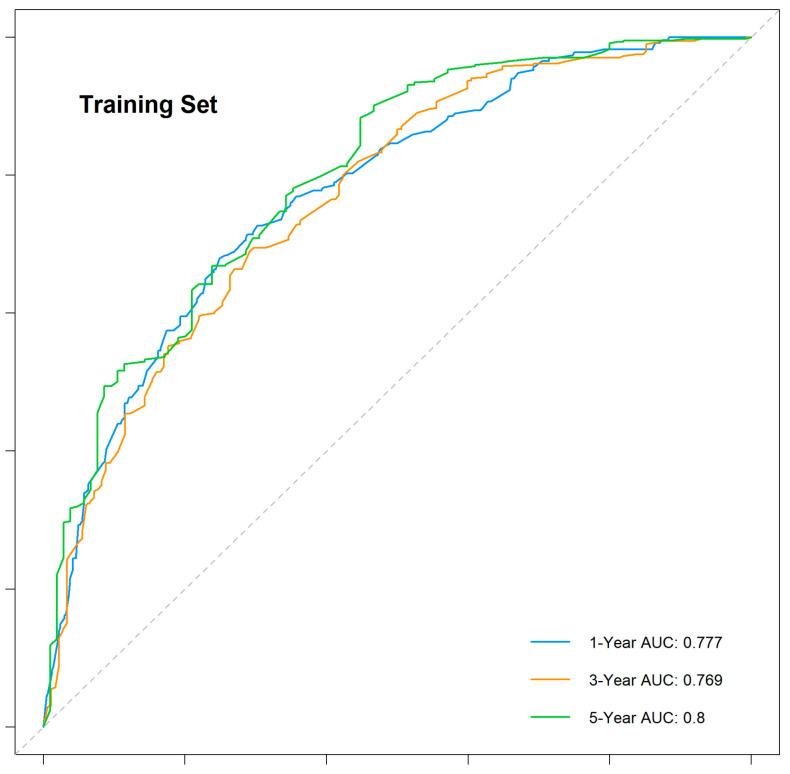
Time-dependent ROC curves for the training cohort.

**Figure 6:**
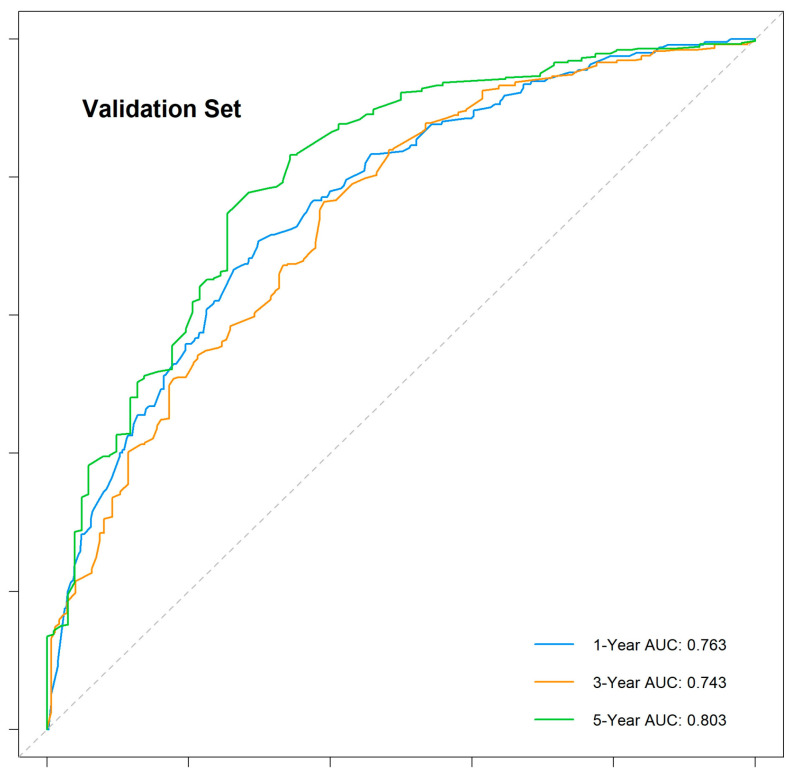
Time-dependent ROC curves for the internal validation cohort.

**Figure 7:**
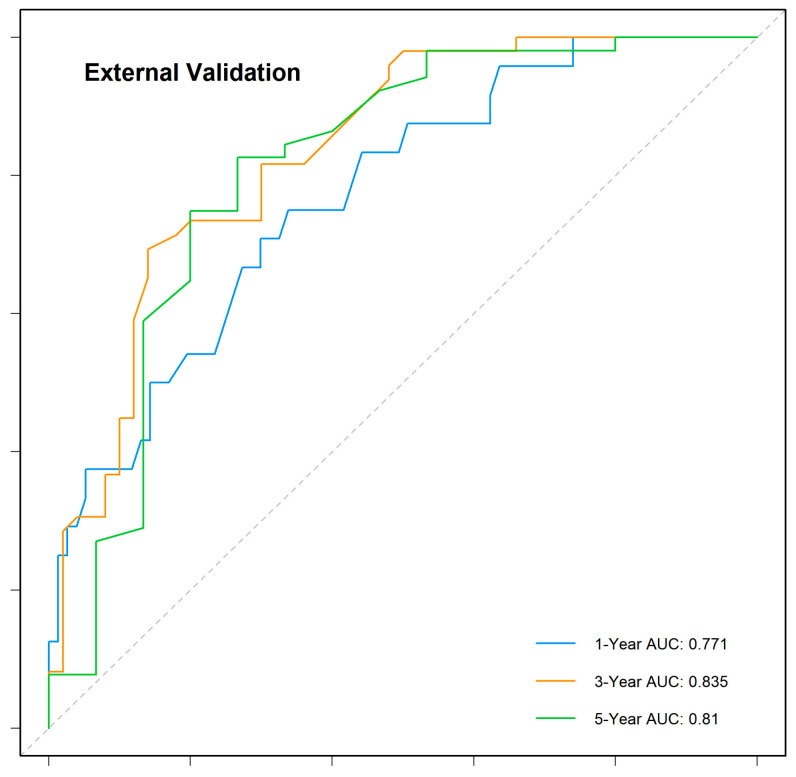
Time-dependent ROC curves for the external validation cohort.

The nomogram demonstrated robust predictive accuracy, with a concordance index (C-index) of 0.767 in the training cohort, 0.798 in the internal validation cohort, and 0.750 in the external validation cohort. Time-dependent receiver operating characteristic (ROC) analysis confirmed that the model outperformed the conventional TNM staging system, with area under the curve (AUC) values of 0.777, 0.769, and 0.800 for 1-, 3-, and 5-year overall survival (OS) in the training cohort (Figure 5). The internal validation cohort showed similar AUC values of 0.763, 0.743, and 0.803 (Figure 6). External validation using the independent Chinese cohort further confirmed the model’s robustness, with AUC values of 0.771, 0.835, and 0.810 (Figure 7).

### 3.6. Calibration Curve Analysis

**Figure 8:**
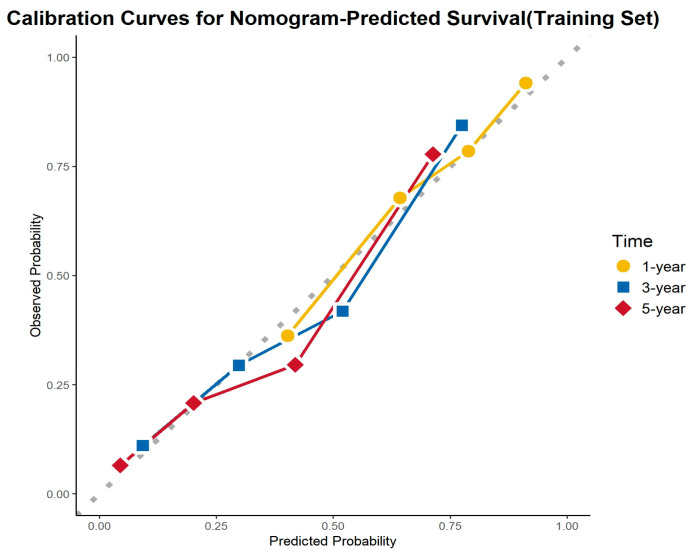
Calibration curves for the training cohort.

**Figure 9:**
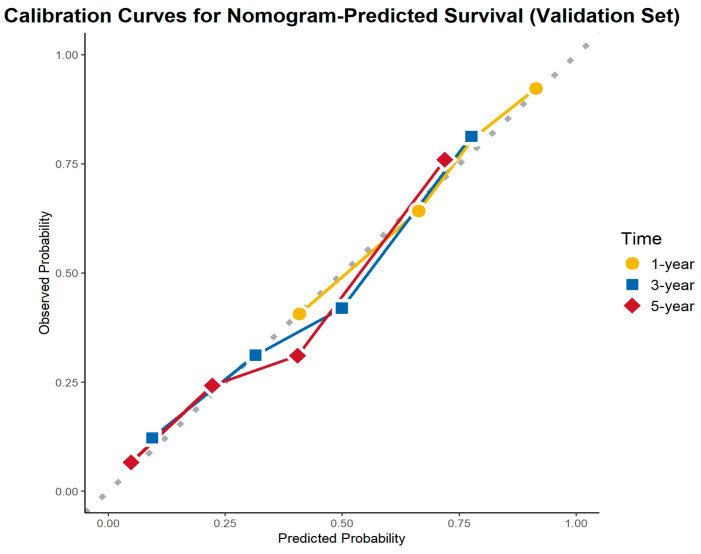
Calibration curves for the internal validation cohort.

**Figure 10:**
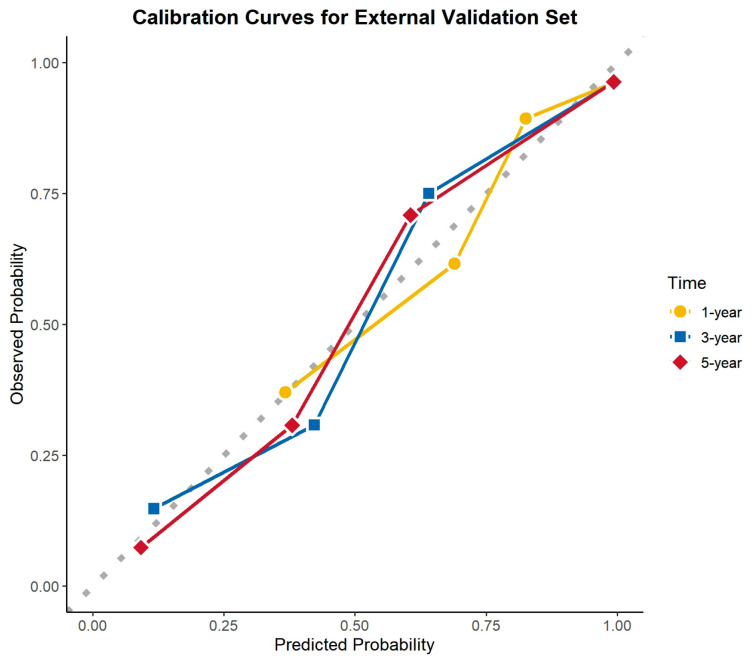
Calibration curves for the external validation cohort.

The calibration curves demonstrated excellent agreement between the predicted and observed survival probabilities across all cohorts and time points. The curves closely aligned with the ideal 45-degree reference line, indicating that the nomogram provided accurate survival predictions. This consistency was observed in the training cohort, internal validation cohort, and external validation cohort for 1-, 3-, and 5-year OS (Figure 8, Figure 9 and Figure 10).

### 3.7. Decision Curve Analysis

**Figure 11:**
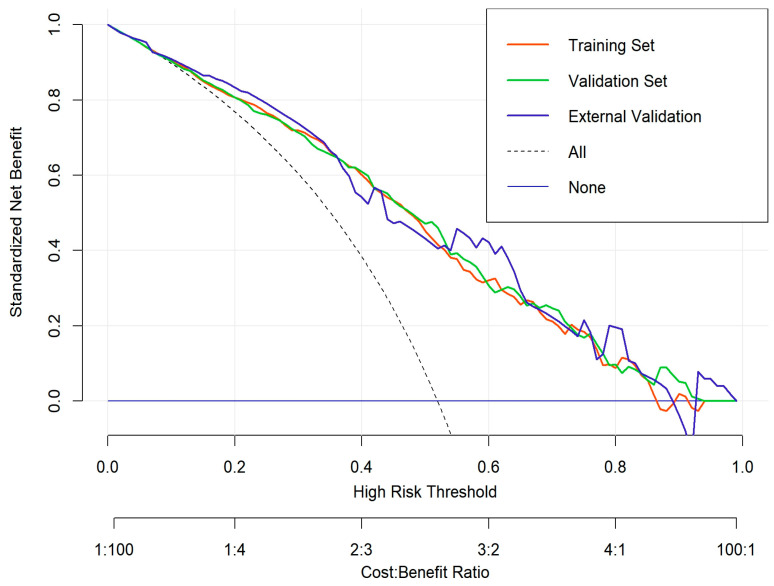
Decision curve analysis (DCA) for the nomogram.

To evaluate the clinical utility of the nomogram, decision curve analysis (DCA) was conducted in the training cohort, internal validation cohort, and external validation cohort (Figure 11). The DCA curves demonstrate the net benefit of using the nomogram for predicting overall survival (OS) across a range of high-risk threshold probabilities. Compared to the treat-all and treat-none strategies, the nomogram consistently provided a higher net benefit across a wide range of threshold probabilities in all three cohorts.

The decision curves for the training (red), internal validation (green), and external validation (blue) cohorts exhibited similar trends, indicating that the nomogram maintains good clinical applicability and generalizability. The model’s performance suggests that it can effectively assist clinicians in making personalized treatment decisions by improving risk stratification and guiding therapeutic interventions for gallbladder cancer patients.

### 3.8. Comparison with TNM Staging System

**Figure 12:**
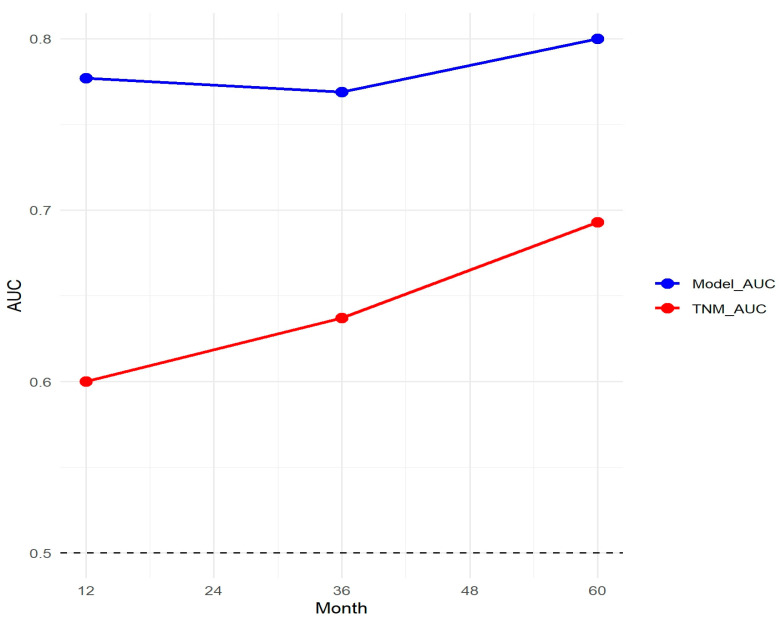
Comparison of the nomogram and TNM staging system.

A comparative analysis of the nomogram and TNM staging system demonstrated the superior predictive capability of the proposed model. The AUC of the nomogram was consistently higher than that of the TNM system across different time points (Figure 12).

### 3.9. Subgroup Analysis of Chemotherapy Benefit

**Figure 13:**
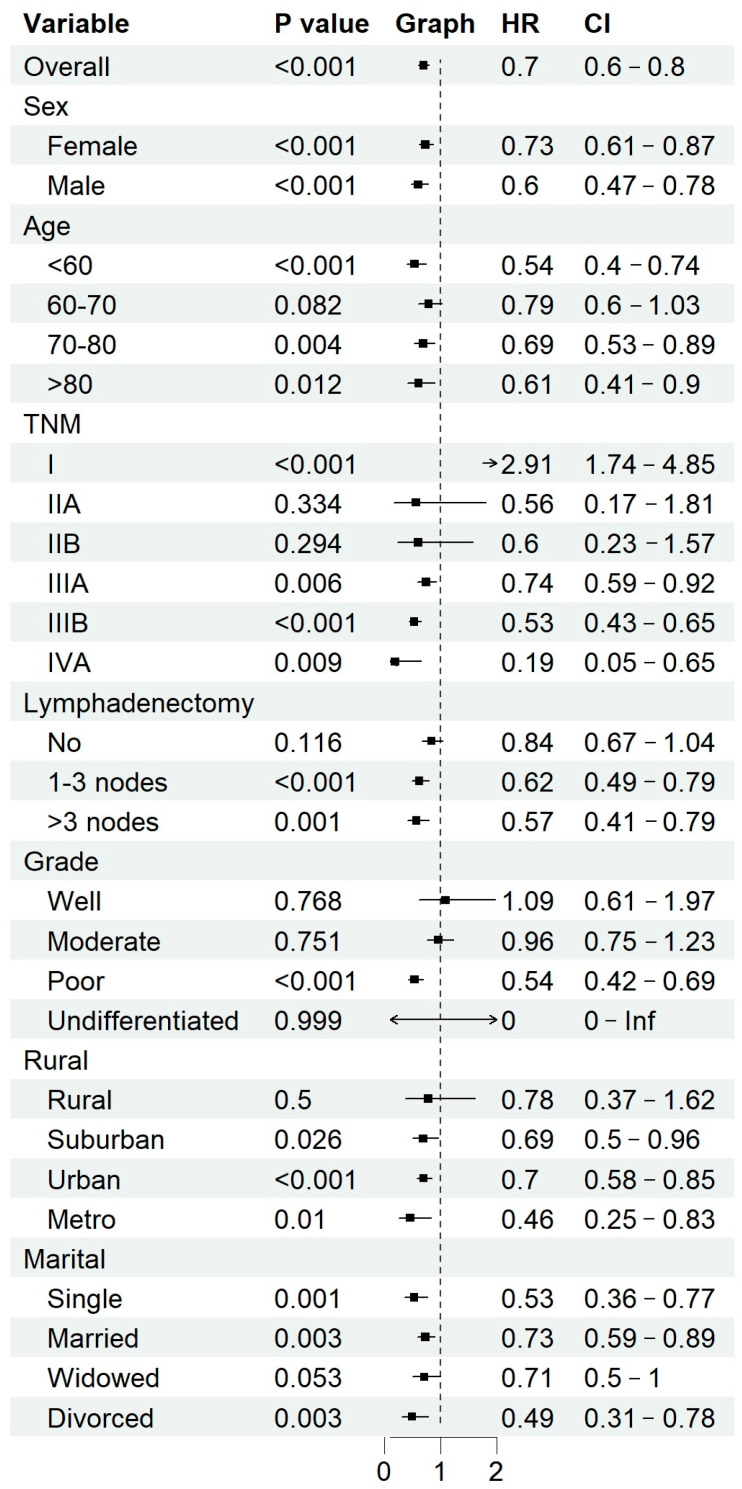
Subgroup analysis of chemotherapy benefits.

A subgroup analysis was conducted to evaluate the impact of adjuvant chemotherapy on overall survival (OS) across different clinical and demographic characteristics (Figure 13). The results demonstrate that chemotherapy significantly improved survival in patients with advanced-stage disease (IIIA, IIIB, IVA) (HR: 0.74, 0.53, and 0.19, respectively; all *p* < 0.01) and poorly differentiated tumors (HR: 0.54, 95% CI: 0.42–0.69, *p* < 0.001). Additionally, patients who underwent lymphadenectomy (1–3 nodes: HR: 0.62; >3 nodes: HR: 0.57, both *p* < 0.01) exhibited a significantly greater chemotherapy benefit compared to those who did not undergo lymph node dissection.

Among patients who did not undergo lymphadenectomy, nearly 47.78% had TNM stage ≤ IIB, suggesting that early-stage disease was a key factor influencing the decision to forgo nodal dissection. However, given the prognostic significance of lymph node involvement, the potential impact of undiagnosed lymph node metastases on treatment decisions remains a concern.

Conversely, early-stage disease (stage I and II) and well- or moderately differentiated tumors did not show a statistically significant survival benefit from chemotherapy. Moreover, younger patients (<60 years old), male patients, and those residing in urban/metropolitan areas appeared to derive greater chemotherapy benefits compared to their respective counterparts. Patients from rural regions and those who were widowed showed fewer clear benefits, potentially reflecting disparities in healthcare access and treatment adherence.

## 4. Discussion

Gallbladder cancer (GBC) is a rare but highly aggressive malignancy, typically diagnosed at advanced stages and associated with poor prognosis [2]. Despite advancements in surgical techniques and multidisciplinary treatments, survival outcomes remain unfavorable, primarily due to late detection and high recurrence rates [4,11,14,20]. In this study, we developed and validated a prognostic nomogram integrating key clinicopathological variables—including age, TNM stage, histological grade, lymphadenectomy status, and chemotherapy—to predict postoperative 1-, 3-, and 5-year survival probabilities. Compared to the conventional TNM staging system, our model demonstrated superior predictive accuracy in both internal and external validation cohorts, supporting its strong clinical applicability.

Previous prognostic studies have often faced limitations, such as small sample sizes [15]. In contrast, our study utilized a large cohort from the SEER database and employed LASSO regression for robust variable selection, effectively minimizing overfitting and enhancing predictive accuracy. The external validation using an independent Chinese cohort further reinforced the model’s reliability and broad applicability across different populations. Our analysis reaffirmed the prognostic significance of TNM stage and tumor differentiation, consistent with the existing literature. Moreover, patients who underwent lymphadenectomy with more than three lymph nodes dissected exhibited significantly improved survival outcomes, supporting current clinical guidelines advocating for lymphadenectomy in GBC [21,22].

The role of postoperative chemotherapy in GBC remains controversial [4,11,23]. Our subgroup analysis revealed that adjuvant chemotherapy significantly improved survival in patients with advanced disease (TNM stage ≥ IIIA) and poorly differentiated tumors, corroborating findings from previous clinical trials. In contrast, patients with TNM stage I disease experienced a detrimental effect from chemotherapy, while those with stage IIA and IIB showed no significant survival difference compared to non-chemotherapy groups. Furthermore, patients who underwent lymphadenectomy derived greater benefit from chemotherapy, whereas those without lymph node dissection did not show a statistically significant advantage.

Additionally, our study observed that patients who did not undergo lymphadenectomy tended to have earlier-stage disease, with 47.78% of these patients classified as TNM stage ≤ IIB. This suggests that the decision to forgo lymph node dissection may be influenced by early tumor stage, possibly due to surgical considerations or perceived lower risk of nodal metastasis. However, given the prognostic significance of lymph node involvement, the absence of lymphadenectomy may lead to understaging, impacting treatment decisions, particularly chemotherapy recommendations. These findings highlight the need to reassess lymphadenectomy strategies and ensure adequate nodal evaluation in surgically resectable gallbladder cancer.

Although our study demonstrated a significant survival benefit of adjuvant chemotherapy in selected patient subgroups, the optimal chemotherapy regimen for gallbladder cancer remains uncertain. Current treatment guidelines for biliary tract cancers, including gallbladder cancer, generally recommend adjuvant chemotherapy with capecitabine [24]. The BILCAP trial suggested a potential benefit of capecitabine adjuvant chemotherapy [11]. A recent randomized trial published in *JAMA Oncology* demonstrated that both gemcitabine plus cisplatin (GC) and concurrent chemoradiotherapy (CCRT) achieved the predefined 1-year DFS endpoint in resected stage II–III gallbladder cancer, suggesting potential benefit in high-risk patients [25].

Not all gallbladder cancer (GBC) patients benefit from chemotherapy, underscoring the need for predictive biomarkers to guide treatment selection. Genetic alterations in key oncogenic pathways, such as TP53, KRAS, and FGFR, have been implicated in GBC progression and may influence chemotherapy responsiveness [26]. Advances in next-generation sequencing (NGS) and single-cell transcriptomics could help define molecular subgroups more likely to benefit from chemotherapy, facilitating precision oncology. Additionally, PD-1/TIM-3 co-expression in the tumor microenvironment is linked to poor prognosis and chemoresistance, while tumor mutational burden (TMB) and microsatellite instability (MSI) are established predictive markers for immunotherapy response, though their prevalence in GBC remains low [27]. The success of checkpoint inhibitors in advanced biliary tract cancers (BTCs) has driven interest in their broader application. The TOPAZ-1 phase III trial demonstrated that durvalumab plus gemcitabine-cisplatin significantly improved overall survival (OS) in advanced BTC [28], providing a rationale for expanding PD-1/PD-L1 inhibitors into the adjuvant setting. Integrating immune checkpoint inhibitors into adjuvant therapy could help mitigate recurrence and improve long-term outcomes in high-risk resected GBC patients. Future studies should focus on biomarker-driven patient selection (e.g., PD-L1 expression, MSI, TMB) to refine immunotherapy strategies in GBC. While chemotherapy remains the current standard, incorporating immune checkpoint inhibitors into postoperative regimens represents a promising strategy for optimizing long-term survival in gallbladder cancer.

Our nomogram surpassed the TNM staging system by incorporating critical prognostic factors such as tumor grade and lymphadenectomy status, leading to higher predictive accuracy (AUC values) at multiple time points and greater clinical utility, as demonstrated by decision curve analysis (DCA). The model consistently provided higher net benefits than conventional staging, reinforcing its potential role in guiding clinical decision-making. Additionally, socioeconomic and geographic disparities were observed in chemotherapy benefits, suggesting that treatment accessibility and adherence may influence survival outcomes. Further efforts should focus on reducing disparities and ensuring equitable access to optimal treatments.

Our study also has certain limitations. The retrospective design introduces potential risks of selection bias and unmeasured confounding variables. Additionally, our analysis did not include molecular biomarkers such as CA19-9, CEA, TP53, KRAS, and FGFR, which have been increasingly recognized as influential in GBC prognosis [29], due to data availability constraints. Future research should integrate molecular and genomic data and explore the use of artificial intelligence and machine learning to further refine personalized prognostic models.

## 5. Conclusions

This study developed and validated a novel postoperative prognostic nomogram for gallbladder cancer, which outperformed the traditional TNM staging system in predicting 1-, 3-, and 5-year OS. Subgroup analysis confirmed the selective benefit of adjuvant chemotherapy, emphasizing its role in advanced-stage and poorly differentiated tumors. Our findings provide a clinically applicable tool for individualized prognosis assessment and treatment decision-making in GBC patients. Future research should focus on multi-center validation, the integration of molecular biomarkers, and further refinement of chemotherapy selection criteria to optimize treatment outcomes.

## Figures and Tables

**Table 1:** Baseline patient characteristics.

	Train	Validation	*p*-Value	SEER (N,%)	Single Center (N, %)
N	924	924		1848	108
Sex			0.44		
Female	646 (69.9)	662 (71.6)		1308 (70.8)	61 (56.5)
Male	278 (30.1)	262 (28.4)		540 (29.2)	47 (43.5)
Age			0.39		
<60	226 (24.46)	239 (25.87)		465 (25.16)	33 (30.56)
60–70	281 (30.41)	269 (29.11)		550 (29.76)	51 (47.22)
70–80	230 (24.89)	254 (27.49)		484 (26.19)	21 (19.44)
>80	187 (20.24)	162 (17.53)		349 (18.89)	3 (2.78)
Race			0.35		
White	665 (71.9)	645 (69.8)		1310 (70.9)	0 (0)
Black	122 (13.2)	113 (12.2)		235 (12.7)	0 (0)
Indian	11 (1.2)	19 (2.1)		30 (1.6)	0 (0)
Asian	114 (12.3)	132 (14.3)		246 (13.3)	108 (100)
Unknown	12 (1.3)	15 (1.6)		27 (1.5)	0 (0)
TNM			0.67		
I	195 (21.1)	195 (21.1)		390 (21.1)	5 (4.6)
IIA	72 (7.8)	86 (9.3)		158 (8.5)	20 (18.5)
IIB	49 (5.3)	61 (6.6)		110 (6.0)	5 (4.6)
IIIA	264 (28.6)	254 (27.5)		518 (28.0)	38 (35.2)
IIIB	332 (35.9)	315 (34.1)		647 (35.0)	34 (31.5)
IVA	12 (1.3)	13 (1.4)		25 (1.4)	6 (5.6)
Lymphadenectomy			0.44		
>3 nodes	202 (21.9)	212 (22.9)		414 (22.4)	73 (67.6)
1–3 nodes	337 (36.5)	354 (38.3)		691 (37.4)	13 (12)
No/Unknown	385 (41.7)	358 (38.7)		743 (40.2)	22 (20.4)
Radiotherapy			0.81		
Yes	172 (18.6)	167 (18.1)		309 (16.7)	4 (3.7)
No/Unknown	752 (81.4)	757 (81.9)		1509 (81.7)	104 (96.3)
Chemotherapy			0.89		
Yes	411 (44.5)	415 (44.9)		826 (44.7)	63 (58.3)
No/Unknown	513 (55.5)	509 (55.1)		1022 (55.3)	45 (41.7)
Grade			0.68		
Well	124 (13.4)	105 (11.4)		229 (12.4)	38 (35.2)
Middle	356 (38.5)	351 (38.0)		707 (38.3)	63 (58.3)
Poorly	268 (29.0)	283 (30.6)		551 (29.8)	7 (6.5)
Undifferentiated	4 (0.4)	5 (0.5)		9 (0.5)	0 (0)
Unknown	172 (18.6)	180 (19.5)		352 (19.0)	0 (0)
Income			1.00		
<75,000	309 (33.4)	309 (33.4)		233 (12.6)	0 (0)
>75,000	117 (12.7)	116 (12.6)		616 (33.3)	0 (0)
Unknown	498 (53.9)	499 (54.00)		999 (54.1)	108 (100)
Marital Status			<0.001		
Married	463 (50.1)	485 (52.5)		948 (53.7)	106 (98)
Single	147 (15.9)	155 (16.8)		302 (17.1)	1 (1)
Widowed	191 (20.7)	87 (9.4)		278 (19.6)	1 (1)
Divorced	82 (8.9)	87 (9.4)		169 (9.6)	0 (0)
Unknown	41 (4.44)	110 (11.90)		151 (8.17)	0 (0)
City			0.59		
Large	578 (62.6)	593 (64.2)		1171 (63.4)	58 (53.7)
Middle	189 (20.5)	168 (18.2)		357 (19.3)	27 (25)
Small	67 (7.3)	59 (6.4)		126 (6.8)	23 (21.3)
Adjacent	48 (5.2)	62 (6.7)		110 (6)	0 (0)
Rural	41 (4.4)	41 (4.4)		82 (4.4)	0 (0)
Unknown	1 (0.1)	1 (0.1)		2 (0.2)	0 (0)

**Table 2:** Multivariable Cox regression analysis of prognostic factors.

Characteristics	Multivariable Analysis for Overall Survival, HR (95% CI)	
**Age**		
<60	1	Reference
60–70	1.75	(0.98–1.41)
70–80	1.53	(1.27–1.83)
>80	2.06	(1.70–2.50)
TNM		
I	1	Reference
IIA	1.36	(0.80–2.30)
IIB	2.20	(1.16–2.30)
IIIA	4.83	(4.39–7.96)
IIIB	7.24	(4.39–11.94)
IVA	7.83	(4.02–15.22)
Lymphadenectomy		
No Dissection/Unknow	1	Reference
1–3 nodes	0.527	(0.448–0.620)
>3 nodes	0.488	(0.403–0.591)
Chemotherapy		
Yes	0.69	(0.601–0.804)
No/Unknow	1	Reference
Grade		
Well	1	Reference
Middle	1.41	(1.11–1.78)
Poorly	2.81	(2.20–3.59)
Undifferentiated	5.08	(2.21–11.67)

## Data Availability

The SEER database used in this study is publicly available and can be accessed at https://seer.cancer.gov/. The external validation data from Tianjin Medical University Cancer Institute and Hospital are not publicly available due to ethical and institutional restrictions.

## References

[1] Siegel R.L., Kratzer T.B., Giaquinto A.N., Sung H., Jemal A. (2025). Cancer statistics, 2025. CA Cancer J. Clin..

[2] Roa J.C., Garcia P., Kapoor V.K., Maithel S.K., Javle M., Koshiol J. (2022). Gallbladder cancer. Nat. Rev. Dis. Prim..

[3] Wang S.J., Fuller C.D., Kim J.S., Sittig D.F., Thomas C.R., Ravdin P.M. (2008). Prediction model for estimating the survival benefit of adjuvant radiotherapy for gallbladder cancer. J. Clin. Oncol..

[4] Lamarca A., Edeline J., McNamara M.G., Hubner R.A., Nagino M., Bridgewater J., Primrose J., Valle J.W. (2020). Current standards and future perspectives in adjuvant treatment for biliary tract cancers. Cancer Treat. Rev..

[5] Feo C.F., Ginesu G.C., Fancellu A., Perra T., Ninniri C., Deiana G., Scanu A.M., Porcu A. (2022). Current management of incidental gallbladder cancer: A review. Int. J. Surg..

[6] Lei S., Huang G., Li X., Xi P., Yao Z., Lin X. (2025). Global Burden, Trends, and Inequalities of Gallbladder and Biliary Tract Cancer, 1990–2021: A Decomposition and Age-Period-Cohort Analysis. Liver Int..

[7] Lazcano-Ponce E.C., Miquel J.F., Munoz N., Herrero R., Ferrecio C., Wistuba I.I., de Ruiz P.A., Urista G.A., Nervi F. (2001). Epidemiology and molecular pathology of gallbladder cancer. CA Cancer J. Clin..

[8] Lv T.R., Wang J.K., Li F.Y., Hu H.J. (2024). Prognostic factors for resected cases with gallbladder carcinoma: A systematic review and meta-analysis. Int. J. Surg..

[9] Amin M.B., Greene F.L., Edge S.B., Compton C.C., Gershenwald J.E., Brookland R.K., Meyer L., Gress D.M., Byrd D.R., Winchester D.P. (2017). The Eighth Edition AJCC Cancer Staging Manual: Continuing to build a bridge from a population-based to a more “personalized” approach to cancer staging. CA Cancer J. Clin..

[10] Chun Y.S., Pawlik T.M., Vauthey J.N. (2018). 8th Edition of the AJCC Cancer Staging Manual: Pancreas and Hepatobiliary Cancers. Ann. Surg. Oncol..

[11] Primrose J.N., Fox R.P., Palmer D.H., Malik H.Z., Prasad R., Mirza D., Anthony A., Corrie P., Falk S., Finch-Jones M. (2019). Capecitabine compared with observation in resected biliary tract cancer (BILCAP): A randomised, controlled, multicentre, phase 3 study. Lancet Oncol..

[12] Balachandran V.P., Gonen M., Smith J.J., DeMatteo R.P. (2015). Nomograms in oncology: More than meets the eye. Lancet Oncol..

[13] Wu J., Zhang H., Li L., Hu M., Chen L., Xu B., Song Q. (2020). A nomogram for predicting overall survival in patients with low-grade endometrial stromal sarcoma: A population-based analysis. Cancer Commun..

[14] Wang S.J., Lemieux A., Kalpathy-Cramer J., Ord C.B., Walker G.V., Fuller C.D., Kim J.S., Thomas C.R. (2011). Nomogram for predicting the benefit of adjuvant chemoradiotherapy for resected gallbladder cancer. J. Clin. Oncol..

[15] Wu Y., Li Q., Cai Z., Zhang Y., Qiu Y., Yang N., Song T., Li S., Lou J., Li J. (2020). Survival prediction for gallbladder carcinoma after curative resection: Comparison of nomogram and Bayesian network models. Eur. J. Surg. Oncol..

[16] Nakachi K., Ikeda M., Konishi M., Nomura S., Katayama H., Kataoka T., Todaka A., Yanagimoto H., Morinaga S., Kobayashi S. (2023). Adjuvant S-1 compared with observation in resected biliary tract cancer (JCOG1202, ASCOT): A multicentre, open-label, randomised, controlled, phase 3 trial. The Lancet.

[17] Emmanuel T., Maupong T., Mpoeleng D., Semong T., Mphago B., Tabona O. (2021). A survey on missing data in machine learning. J. Big Data.

[18] Wyss R., van der Laan M., Gruber S., Shi X., Lee H., Dutcher S.K., Nelson J.C., Toh S., Russo M., Wang S.V. (2024). Targeted learning with an undersmoothed LASSO propensity score model for large-scale covariate adjustment in health-care database studies. Am. J. Epidemiol..

[19] Tang G., Qi L., Sun Z., Liu J., Lv Z., Chen L., Huang B., Zhu S., Liu Y., Li Y. (2021). Evaluation and analysis of incidence and risk factors of lower extremity venous thrombosis after urologic surgeries: A prospective two-center cohort study using LASSO-logistic regression. Int. J. Surg..

[20] Catalano G., Alaimo L., Chatzipanagiotou O.P., Ruzzenente A., Aucejo F., Marques H.P., Lam V., Hugh T., Bhimani N., Maithel S.K. (2024). Machine learning prediction of early recurrence after surgery for gallbladder cancer. Br. J. Surg..

[21] Liu Z.P., Guo W., Yin D.L., Chen W.Y., Wang J.Y., Li X.L., Yue P., Yu C., Wu Z.P., Ding R. (2023). Textbook outcomes in liver surgery for gallbladder cancer patients treated with curative-intent resection: A multicenter observational study. Int. J. Surg..

[22] Shirai Y., Wakai T., Sakata J., Hatakeyama K. (2012). Regional lymphadenectomy for gallbladder cancer: Rational extent, technical details, and patient outcomes. World J. Gastroenterol..

[23] Ozer M., Goksu S.Y., Sanford N.N., Porembka M., Khurshid H., Ahn C., Maxwell M.C., Beg M.S., Kazmi S.M. (2022). A Propensity Score Analysis of Chemotherapy Use in Patients with Resectable Gallbladder Cancer. JAMA Netw. Open.

[24] Vogel A., Bridgewater J., Edeline J., Kelley R.K., Klumpen H.J., Malka D., Primrose J.N., Rimassa L., Stenzinger A., Valle J.W. (2023). Biliary tract cancer: ESMO Clinical Practice Guideline for diagnosis, treatment and follow-up. Ann. Oncol..

[25] Ostwal V., Patkar S., Engineer R., Parulekar M., Mandavkar S., Bhargava P., Srinivas S., Krishnatry R., Gudi S., Kapoor A. (2024). Adjuvant Gemcitabine Plus Cisplatin and Chemoradiation in Patients with Gallbladder Cancer: A Randomized Clinical Trial. JAMA Oncol..

[26] Song X., Hu Y., Li Y., Shao R., Liu F., Liu Y. (2020). Overview of current targeted therapy in gallbladder cancer. Signal Transduct. Target. Ther..

[27] He X., Peng Y., He G., Ye H., Liu L., Zhou Q., Shi J., Fu S., Wang J., Zhou Z. (2023). Increased co-expression of PD1 and TIM3 is associated with poor prognosis and immune microenvironment heterogeneity in gallbladder cancer. J. Transl. Med..

[28] Oh D.Y., He A.R., Bouattour M., Okusaka T., Qin S., Chen L.T., Kitano M., Lee C.K., Kim J.W., Chen M.H. (2024). Durvalumab or placebo plus gemcitabine and cisplatin in participants with advanced biliary tract cancer (TOPAZ-1): Updated overall survival from a randomised phase 3 study. Lancet Gastroenterol. Hepatol..

[29] Wen Z., Si A., Yang J., Yang P., Yang X., Liu H., Yan X., Li W., Zhang B. (2017). Elevation of CA19-9 and CEA is associated with a poor prognosis in patients with resectable gallbladder carcinoma. HPB.

